# Lipid peroxidation products induce carbonyl stress, mitochondrial dysfunction, and cellular senescence in human and murine cells

**DOI:** 10.1111/acel.14367

**Published:** 2024-10-11

**Authors:** T. Blake Monroe, Ann V. Hertzel, Deborah M. Dickey, Thomas Hagen, Simon Vergara Santibanez, Islam A. Berdaweel, Catherine Halley, Patrycja Puchalska, Ethan J. Anderson, Christina D. Camell, Paul D. Robbins, David A. Bernlohr

**Affiliations:** ^1^ Department of Biochemistry, Molecular Biology and Biophysics University of Minnesota‐Twin Cities Minneapolis Minnesota USA; ^2^ Department of Pharmaceutical Sciences & Experimental Therapeutics, College of Pharmacy, Fraternal Order of Eagles Diabetes Research Center University of Iowa Iowa City Iowa USA; ^3^ Department of Medicine University of Minnesota‐Twin Cities Minneapolis Minnesota USA; ^4^ Institute for the Biology of Aging and Metabolism University of Minnesota‐Twin Cities Minneapolis Minnesota USA; ^5^ Present address: Department of Clinical Pharmacy and Pharmacy Practice, College of Pharmacy Yarmouk University Irbid Jordan

**Keywords:** 4‐HNE, adipose, lipid peroxidation, mitochondrion, senescence

## Abstract

Lipid enals are electrophilic products of lipid peroxidation that induce genotoxic and proteotoxic stress by covalent modification of DNA and proteins, respectively. As lipid enals accumulate to substantial amounts in visceral adipose during obesity and aging, we hypothesized that biogenic lipid enals may represent an endogenously generated, and therefore physiologically relevant, senescence inducers. To that end, we identified that 4‐hydroxynonenal (4‐HNE), 4‐hydroxyhexenal (4‐HHE) or 4‐oxo‐2‐nonenal (4‐ONE) initiate the cellular senescence program of IMR90 fibroblasts and murine adipose stem cells. In such cells, lipid enals induced accumulation of γH2AX foci, increased p53 signaling, enhanced expression of p21^Cip1^, and upregulated the expression and secretion of numerous cytokines, chemokines, and regulatory factors independently from NF‐κB activation. Concomitantly, lipid enal treatment resulted in covalent modification of mitochondrial proteins, reduced mitochondrial spare respiratory capacity, altered nucleotide pools, and increased the phosphorylation of AMP kinase. Lipid‐induced senescent cells upregulated *BCL2L1* (Bcl‐xL) and *BCL2L2* (Bcl‐w). and were resistant to apoptosis while pharmacologic inhibition of BAX/BAK macropores attenuated lipid‐induced senescence. In situ, the 4‐HNE scavenger L‐carnosine ameliorated the development of the cellular senescence, while in visceral fat of obese C57BL/6J mice, L‐carnosine reduced the abundance of 4‐HNE‐modified proteins and blunted the expression of senescence biomarkers *CDKN1A* (p21^Cip1^), *PLAUR*, *BCL2L1*, and *BCL2L2.* Taken together, the results suggest that lipid enals are endogenous regulators of cellular senescence and that biogenic lipid‐induced senescence (BLIS) may represent a mechanistic link between oxidative stress and age‐dependent pathologies.

## Graphical abstract

Lipid enals covalently modify mitochondrial proteins and induce genotoxic stress initiating the cellular senescence program in IMR90 and adipose stem cells leading to expression of p53, p21, and SASP factors.

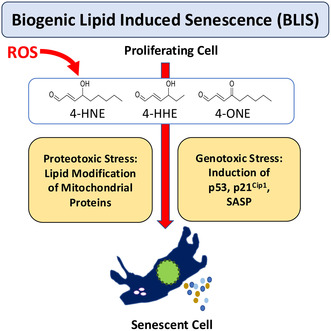

**Abbreviations**
4‐HHE: 4‐hydroxy 2,3 hexenal
4‐HNE: 4‐hydroxy 2,3 nonenal
4‐MU: 4‐methylumbelliferone
4‐ONE: 4‐oxo‐2,3 nonenal
ALCAT1: Acyl‐CoA:lysocardiolipin acyltransferase‐1
ANGPTL4: Angiopoietin‐like 4
AQP1: Aquaporin 1
BAK: BCL2 antagonist/killer
BAX: BCL2 associated X
BCA: Bicinchoninic acid
BCB: Bax channel blocker
BCL2: B‐cell lymphoma 2
BCL2L1: BCL2 Like 1
BCL2L2: BCL2 Like 2
BIPv5: Bax inhibitor peptide V5
BLIS: Biogenic lipid‐induced senescence
BMP3: Bone morphogenetic protein 3
BSA: Bovine serum albumin
C_12_FDG: 5‐Dodecanoylaminofluorescein Di‐β‐D‐Galactopyranoside
CAR T: Chimeric antigen receptor T‐cell
CDKN1A: Cyclin‐dependent kinase inhibitor 1A
CDKN2A: Cyclin‐dependent kinase inhibitor 2A
CDNF: Cerebral dopamine neurotrophic factor
cGAS: Cyclic GMP‐AMP Synthase
CHAPS: 3‐[(3‐cholamidopropyl)dimethylammonio]‐1‐propanesulfonate
COXIV: Cytochrome c oxidase subunit 4
CPT1A: Carnitine palmitoyltransferase I
CTF1: Cardiotrophin‐1
CXCL1: Chemokine ligand 1
CXCL14: Chemokine ligand 14
D_2_O: Deuterium oxide
DAVID: Database for annotation, visualization and integrated discovery
DKK2: Dickkopf‐related protein 2
DMEM: Dulbecco's Modified Eagle Medium
EGFL7: EGF‐like domain‐containing protein 7
ELISA: Enzyme‐linked Immunosorbent Assay
FCCP: Carbonyl cyanide‐p‐trifluoromethoxyphenylhydrazone
FGF2: Fibroblast growth factor 2
FITC: Fluorescein isothiocyanate
GAPDH: Glyceraldehyde 3‐phosphate dehydrogenase
Gst10: GST C‐terminal domain‐containing protein
GstA4: Glutathione S‐Transferase Alpha 4
GTT: Glucose Tolerance Test
HADHB: Hydroxyacyl‐CoA Dehydrogenase Trifunctional Multienzyme Complex Subunit Beta
HFHS: High fat high sucrose
HMGB1: High mobility group box 1
IGFBP3: Insulin‐like growth factor binding protein 3
IGFBP7: Insulin‐like growth factor binding protein 7
IL‐1β: Interleukin 1β
IL6: Interleukin 6
IL8: Interleukin 8
INA: Internexin Neuronal Intermediate Filament Protein Alpha
IRF3: Interferon Response Factor 3
ITT: Insulin Tolerance Test
KEGG: Kyoto Encyclopedia of Genes and Genomes
KISS1: Kisspeptins
KRT34: Keratin 34
LCLAT1: Lysocardiolipin acyltransferase 1
LIPG: Lipase G
MEK: Mitogen‐activated protein kinase kinase
MEM: Minimum Essential Media
MFAP5: Microfibril‐associated glycoprotein 5
MiDAS: mitochondrial dysfunction associated senescence
miMOMP: minority mitochondrial outer membrane permeabilization
MMP3: Matrix metalloproteinase‐3
mtDNA: Mitochondrial DNA
mtMOMP: mitochondrial outer membrane permeabilization
MUG: 4‐methylumbelliferyl‐β‐d‐galactopyranoside
NF‐κB: Nuclear factor kappa B
OSTN: Osteocrin
PLA2G15: Phospholipase A2 group XV
PLAUR: Urokinase plasminogen activator
PRDX6: Peroxyredoxin 6
PVDF: Polyvinylidene difluoride
REN: Renin
RIPA: Radio‐Immunoprecipitation Assay
SASP: Senescence associated secretory phenotype
SA‐β‐gal: Senescence associated β galactosidase
SEMA3E: Semaphorin 3E
SERPINE1: Serine protease inhibitor E1
SERPINI1: Serine protease inhibitor I1
SnC: Senescent cell
STING: Stimulator of Interferon Genes
SVF: Stromal Vascular Fraction
TFEA: Transcription Factor Enrichment Analysis
Tfiie: Transcription factor II E
TIMP1: Tissue inhibitors of metalloproteinase 1
TUNEL: Terminal deoxynucleotidyl transferase dUTP nick end labeling
ULBP2: UL16 binding protein 2
VAMP7: Vesicle Associated Membrane Protein 7
WNT16: Wnt Family Member 16
X‐gal: 5‐Bromo‐4‐chloro‐1H‐indol‐3‐yl β‐D‐galactopyranoside
γH2AX: H2A histone family member X phosphorylated at Ser 139

## 1 INTRODUCTION

Cellular senescence is a cell fate characterized by coincident set of features such as cell cycle arrest, enhanced expression of cyclin‐dependent kinase inhibitors, resistance to apoptosis, and secretion of a battery of proteins (cytokines, chemokines, protease inhibitors) collectively referred to as the senescence‐associated secretory phenotype (SASP). Senescent cells (SnCs) accumulate in tissue over time and, with their inflammatory paracrine effects of the SASP and associated stem cell exhaustion, are thought to contribute to dysfunction associated with age. Transgenic mouse models utilizing systems that allow for selective elimination of cells highly expressing p16^ink4a^ or p21^Cip1^ have revealed an association between SnC accumulation in adipose and age‐related metabolic disease (Xu, Palmer, et al., 2015). Recent evidence demonstrates a link between senescence in adipose depots during obesity and the development of systemic insulin resistance (Suda et al., 2021; Wang et al., 2022). Using mouse models of obesity coupled with xenografts of human adipose, ablation of p21^Cip1^ positive cells in adipose tissue, through either pharmacological or genetic means, alleviated the development of metabolic dysfunction (Wang et al., 2022).

While accumulation of SnCs in visceral adipose tissue is a causal factor in age‐ and obesity‐associated metabolic dysfunction (Suda et al., 2021; Wang et al., 2022), the molecular mechanisms that initiate the senescence program in fat have remained enigmatic and largely unexplored. Known senescence inducers include gamma radiation, H_2_O_2_, hypoxia, rotenone, and telomeric attrition (Chen et al., 2001). Oxidative stress is frequently a correlate to cellular senescence and leads to the peroxidation of membrane phospholipids and triglycerides (Catalá, 2009) resulting in chemical production of lipid enals such as 4‐hydroxynonenal, 4‐hydroxyhexenal, and 4‐oxo‐2‐nonenal. In murine models of metabolic and genetic obesity, 4‐HNE and 4‐ONE accumulate in adipose tissue to the highest know levels (approaching milligram/gm quantities) in visceral, but not subcutaneous adipose depots (Long et al., 2013). Moreover, expression of the biogenic aldehyde detoxification enzymes glutathione S‐transferase, peroxyredoxin, glutathione peroxidase, and aldehyde dehydrogenase are down‐regulated in visceral, but not subcutaneous, adipose tissue and cultured adipocytes in response to cytokine treatment, promoting the accumulation of 4‐HNE (Curtis et al., 2010). Importantly, comparison of lifespan extension across multiple murine platforms identified GstA4, the major 4‐HNE detoxification enzyme, as a key determinant of longevity (Ayyadevara et al., 2007). Consistent with this, deletion of *Caenorhabditis elegans Gst10* decreased lifespan while overexpression of either *C. elegans Gst10* or murine Gsta4 into *Gst10* null animals increased lifespan (Swiader et al., 2021).

Due to their chemical solubility, lipid enals readily diffuse across phospholipid bilayers (Vazdar et al., 2012), allowing them to serve as paracrine and autocrine regulators of senescence. In their capacity as reactive electrophiles, lipid enals covalently modify nucleophilic species including those on nucleic acids and proteins via Michael addition and Schiff base formation. This modification is termed carbonyl stress and has been shown to typically, but not always, represent a loss‐of‐function in both DNA and proteins (Yoval‐Sánchez & Rodríguez‐Zavala, 2012). Since lipid enals are more stable than reactive oxygen species such as hydrogen peroxide or superoxide, they are longer‐lived and therefore represent potential mediators of senescence in vivo (Siems & Grune, 2003). Even though bioactive lipids are implicated in a variety of pathologies (Das, 2021; Wiley et al., 2021) and lipid peroxidation is recognized as an index and effector of oxidative stress, lipid enals themselves have not been investigated in detail as mediators of cellular senescence. Herein we describe experiments in vitro and in vivo that demonstrate reactive lipid enals induce cellular senescence via a bifurcated process termed Biogenic Lipid‐Induced Senescence (BLIS) involving both DNA damage and mitochondrial dysfunction. Moreover, the aldehyde quenching molecule L‐ carnosine attenuates BLIS both in vitro and in vivo, suggesting a novel therapeutic opportunity to affect senescence by targeting lipid peroxidation products.

## 2 METHODS AND PROTOCOLS

### 2.1 Cell culture

IMR90 human lung fibroblasts (ATCC CCL 186) were cultured at 37°C, 5% CO_2_ in Minimum Essential Medium (Thermo 11095080), supplemented with fetal bovine serum (10%, Atlas Biologicals FS‐0500‐AD), sodium pyruvate (1%, Thermo 11360070), and MEM nonessential amino acids (1%, Thermo 11140050). All IMR90s used for experiments were between passages 2 and 6. Murine adipose progenitor cells in the stromal vascular fraction (SVF) were isolated from murine adipose tissue by digestion in collagenase and differential plating as previously described (Xu, Hertzel, et al., 2015). Murine SVF cells were then cultured in DMEM (Thermo 10569–100) with 10% FBS and used for experiments in passages 2 and 3. Except where otherwise noted, BLIS was induced by treating cells continuously with either 20 μM 4‐hydroxynonenal (Cayman 32100), 20 μM of 4‐hydroxyhexenal (Cayman 32060), or 10 μM of 4‐oxo‐2‐nonenal (Cayman 10185). The concentration of lipid used to induce BLIS for each enal was determined experimentally over a range of lipid levels and assessed by cell viability and β‐galactosidase levels. Continuous treatment entails never aspirating media from cells, and instead adding fresh media every 3 days.

### 2.2 Mouse model of diet induced obesity and L‐carnosine treatment

All interventions and procedures were reviewed and approved by the University of Iowa Institutional Animal Care and Use Committee or the University of Minnesota Institutional Animal Care and Use Committee prior to beginning the study. Male C57BL/6J mice were obtained from Jackson Laboratory at ~4 weeks of age. At 8–10 weeks, mice (*n* = 10) were randomly assigned to either normal chow diet (Control, D20122207, Research Diets, Inc) or high fat high sucrose diet (HFHS), (D09071704, Research Diets, Inc) for 16 weeks. Protein and macronutrient composition in each diet were matched except that the HFHS diet consisted of lard‐based fat (35.5% daily kcal) and cholesterol (1.5% daily kcal), and carbohydrate consisted of sucrose (38% daily kcal), while the Control diet consisted of low fat (6% daily kcal), and starch‐based carbohydrates (~75% daily kcal). Seven weeks after starting the diets, a subset of the mice on HFHS diet began receiving L‐carnosine supplemented in their drinking water (80 mM). Both the HFHS diet and carnosine‐supplemented water were refreshed every 3–4 days.

### 2.3 Metabolic parameters

To assess the impact of dietary L‐carnosine on glycemic control in the mice, intraperitoneal glucose (GTT) and insulin tolerance tests (ITT) were performed at 14 and 15 weeks after starting the diet, respectively, using standard techniques. Briefly, for GTT a dose of 1 g/kg of 50% dextrose in saline, and for ITT a dose of 0.75 U/kg insulin in saline, were administered via intraperitoneal injection after 6 h of fasting, followed by blood glucose measurements from the tail stick at subsequent timepoints using a glucometer (OneTouch, Verio Flex).

### 2.4 Mitochondrial isolation

Mitochondria were isolated from cells using a reagent‐based method following manufacturer's instructions (Thermo 89,874). Briefly, cells were lysed in proprietary buffers, nuclei and unlysed cells were removed by low‐speed (700 × *g*) centrifugation, and mitochondria were pelleted by 12,000 × g centrifugation, with the resulting supernatant constituting the cytosolic fraction of the cellular extract. Identity of mitochondrial and cytosolic fractions were confirmed by probing for cytochrome c oxidase subunit 4 (COXIV) and mitogen‐activated protein kinase kinase (MEK).

### 2.5 Carbonyl detection

Cells were lysed in coupling buffer (100 mM sodium acetate, 20 mM NaCl, 0.1 mM EDTA, pH 5.5) supplemented with 1% Triton and protease inhibitors. Cell extracts were then incubated with EZ‐link biotin hydrazide (5 mM) for 2 h at room temperature to add biotin groups to carbonyl moieties. Functionalized samples were then resolved by SDS‐PAGE and transferred to a PVDF membrane. Membranes were blocked overnight at 4°C in Intercept Blocking Buffer (LI‐COR 927–60,001), incubated with IRDye CW800 streptavidin (LI‐COR 926–32,230), rinsed, and imaged using a LI‐COR Odyssey imager.

### 2.6 Mass spectrometric measurement of 4‐hydroxynonenal

4‐hydroxynonenal was quantitated from frozen adipose tissue using the method described by Wang et al., 2012. Briefly, frozen adipose samples were homogenized in ice‐cold phosphate buffered saline: water (1/10, v/v) at 4°C and the protein level was quantitated using the bicinchoninic acid method. 0.7 nmol of 4‐hydroxynonenal‐d_3_ (Cayman) per 1 mg of protein was added to the homogenate as an internal standard. Homogenate with internal standard was extracted using the modified Bligh and Dyer extraction method, dried, and diluted in chloroform/methanol (1/1, v/v). Subsequently, 20 μL of extract was mixed with 22 μL of 125 mM carnosine, 250 μL of water, and incubated at 37°C for 24 h. for derivatization (Wang et al., 2012). Derivatization was ended by adding 1.5 mL of chloroform/methanol (1/1, v/v) and 0.5 mL water and derivatized samples extracted into the upper aqueous layer after centrifugation at 2700 rpm for 5 min. The collected aqueous phase was washed two times by addition of chloroform (0.75 mL) and evaporated under a nitrogen stream. The residue was reconstituted in 100 μL of water/methanol (1/1, v/v), diluted 20‐fold in methanol/water/formic acid (80/20/0.1, v/v/v) and infused in TSQ Quantis mass spectrometer (Thermo, San Jose, CA) equipped with an automated nanospray device (Triversa Nanomate, Advion Bioscienc Ltd. Ithaca, NY). Analysis was performed in positive ion mode using neutral loss scans of 71.2 and 117.2 with collision energies 23 and 28 eV, respectively, and collision gas pressure at 1 mTorr. Quantitation of 4‐hydroxynonenal species was obtained by averaging the results determined from neutral loss scans of 71.2 and 117.2 in comparison to internal standard using in‐house software.

### 2.7 Western blotting

Proteins from cell extracts in RIPA buffer were resolved by SDS‐PAGE, transferred to an Immobilon‐P PVDF membrane (Millipore IPVH00010) at 4°C, blocked for 1 h at room temperature with Intercept Blocking Buffer, and then incubated with primary antibody to desired target overnight. All antibodies used are listed in Table S1. Membranes were then rinsed in tris‐buffered saline buffer with 1% Tween, incubated for 1 h at room temperature with fluorescently‐ or near infrared‐labeled secondary antibodies (LI‐COR, IRDye 680RD or 800CW), and imaged using a LI‐COR Odyssey Imager or an iBright CL1500 Imaging System (Thermo).

### 2.8 Enzyme‐linked immunosorbent assay (ELISA)

To quantitate secreted IGFBP3 and MMP3, ELISAs were performed on conditioned media using commercially available kits (Biotechne DGB300, DMP300), according to manufacturer's instructions. Briefly, conditioned media from IMR90s treated for a week with vehicle or lipid enals were loaded into clear, plastic 96‐well plates provided with the kits. After an hour of antigen adhesion, plates were rinsed and probed with provided primary and secondary antibodies, chromogenic substrate was added to samples, and absorbance was measured on a Synergy 4 Reader (Agilent).

### 2.9 Senescence‐associated β‐galactosidase staining

Chromogenic staining for senescence‐associated β‐galactosidase (SA‐β‐gal) was performed with galactosidase substrate 5‐Bromo‐4‐chloro‐1H‐indol‐3‐yl β‐D‐galactopyranoside (X‐gal, Sigma 16,555) as previously described (Itahana et al., 2007). Briefly, IMR90s subjected to BLIS were fixed with paraformaldehyde/glutaraldehyde (2%/0.1%) for 5 min at room temperature. Fixed cells were then rinsed with phosphate‐buffered saline and stained with 1 mg/mL X‐gal in a citrate/phosphate buffer (5 mM Potassium Ferricyanide, 5 mM Potassium Ferrocyanide, 2 mM MgCl_2_, 150 mM NaCl, 40 mM citric acid, 40 mM sodium phosphate, pH 6) for 4 h at 37°C, protected from light. Stained cells were then rinsed and visualized on an Olympus CK40 microscope.

The conversion of fluorogenic galactosidase substrate 4‐methylumbelliferyl‐β‐d‐galactopyranoside (MUG, Sigma M1633) to fluorophore 4‐methylumbelliferone (4‐MU) was used to quantify SA‐β‐gal in cell lysate previously described (Gary & Kindell, 2005). Briefly, cells were washed with PBS and lysed in CHAPS lysis buffer (5 mM 3‐[(3‐cholamidopropyl)dimethylammonio]‐1‐propanesulfonate, 40 mM citric acid, 40 mM sodium phosphate, pH 6) supplemented with protease inhibitors. Samples were kept on ice for an hour, vortexed, and centrifuged at 12,000 × g for 5 min. Total protein in each sample was measured by BCA assay. Samples were combined with a 2X reaction buffer (40 mM citric acid, 40 mM sodium phosphate, 10 mM β‐mercaptoethanol, 4 mM MgCl2, pH 6), and MUG (final concentration 1.7 mM). Mixtures were incubated at 37°C for 1 h, after which reactions were terminated with 400 mM sodium carbonate. Fluorescence was then measured using a plate reader at excitation/emission wavelengths of 360 nm/465 nm.

Generation of a fluorophore from SA‐β‐gal substrate 5‐Dodecanoylaminofluorescein Di‐β‐D‐Galactopyranoside (C_12_FDG) was used to visualize SA‐β‐gal activity and quantify SnCs as previously described (Fuhrmann‐Stroissnigg et al., 2019). IMR90s treated with a vehicle control, lipid enals, or etoposide were seeded (5000 cells/well) on a 96‐well plate (Corning, 3614). All cells were treated with bafilomycin (100 nM) for 1 h to alkalinize lysosomes. C_12_FDG (10 μM) was then added and cells were incubated for 2 h. Cells were then washed and nuclei were counterstained with Hoechst 33342 before imaging using a Cytation 1 (Agilent). SnCs were quantified using C_12_FDG fluorescence intensity thresholds determined by the etoposide‐treated group.

### 2.10 Cell proliferation assay

Cellular proliferation was assessed by a commercially available kit (CyQUANT, Thermo C7026) using the manufacturer's instructions. Briefly, cells treated as described were lysed and then incubated with a proprietary DNA‐binding fluorogenic probe (CyQUANT GR dye). Fluorescence of each sample was then determined on a plate reader using excitation/emission wavelengths of 485/528 nm. A standard curve was generated using serial dilutions of pellets with known cell number quantities as determined by an automated cell counter (Countess 3), and cell number in samples were quantified by interpolating on the standard curve.

### 2.11 Metabolomic analysis

Polar metabolites were extracted from cells using a chloroform‐methanol extraction procedure. Briefly, cells were lysed by mechanical shearing in a 28‐gauge needle in 300 μL 1% sodium chloride solution. 250 μL of lysate was added to 500 μL chloroform and 250 μL methanol and the mixture was vortexed and centrifuged at 13,000 × *g* for 10 min. The upper aqueous/methanol layer was removed to a new tube and the chloroform layer was washed with 250 μL of 1:1 1% sodium chloride‐methanol. Three milliliters of methanol were added to the aqueous phase to facilitate drying under nitrogen and stored at −80°C until nuclear magnetic resonance (NMR) analysis. Immediately prior to the NMR analysis, the dried film was resuspended in 100 mM sodium phosphate in D_2_O (pH 7.4) containing 20 μM trimethylsilyl propionic acid as a calibration standard with a volume that ensured equal loading across all samples.

### 2.12 Real time measurement of oxygen consumption

Oxygen consumption rate and extracellular acidification rate of IMR90s were measured using a Mito Stress Test Kit (Agilent 103,015–100) and a Seahorse XFe24 Analyzer. IMR90s were treated as described and seeded (9 × 10^4^ cells/well) the day before the assay. An hour before the assay, IMR90s were cultured in unbuffered DMEM for 1 h. at 37°C at atmospheric CO_2_. Plated cells and a loaded cassette containing oligomycin (1 μM), FCCP (1.5 μM), and rotenone (1 μM)/antimycin A (1 μM) were placed into the analyzer and the assay was performed following manufacturer's instructions. OCR is measured in triplicate at baseline and after each added pharmacological agent.

### 2.13 γH2AX foci visualization

H2AX phosphorylated at Ser 139 (γH2AX) was visualized using immunofluorescent microscopy. IMR90s seeded onto coverslips were treated as described and fixed with a 4% paraformaldehyde solution for 12 min. Fixed samples were then permeabilized with 0.2% Triton‐X and blocked with 5% BSA for 1 h. at room temperature. Samples were incubated in primary antibody (1:200) overnight at 4°C. After rinsing, they were incubated with secondary antibody for 1 h. at room temperature. Coverslips were rinsed again, mounted onto slides, and imaged using a Leica Fluorescence Microscopy system (DM5500B).

### 2.14 Real time quantitative polymerase chain reaction (RT‐qPCR)

RNA was isolated using TRIzol reagent (Invitrogen 15,596,026) and after extraction, precipitation, and resuspension, the RNA was subjected to iScript reverse transcriptase (Bio‐Rad, 1,708,891) to synthesize cDNA. Real time quantitative PCR (RT‐qPCR) assays were then performed using SYBR green dye on a Bio‐Rad CFX96 Real Time System. Genes used as internal controls for human and mouse samples were VAMP7 and TFIIE, respectively. Sequences for all primers used can be found in Table S2.

### 2.15 RNA sequencing and analysis

Libraries were synthesized from equal amounts of high‐quality mRNA (1 μg) from IMR90s treated for 1 week with vehicle or 4‐HNE. These libraries were then sequenced using a Lumina NovaSeq 6000. 2 × 150 bp FastQ paired end reads for 6 samples (*n* = 85.3 Million average reads per sample) were trimmed using Trimmomatic (v 0.33) enabled with the optional “‐q” option; 3 bp sliding‐window trimming from 3′ end requiring minimum Q30. Quality control on raw sequence data for each sample were performed with FastQC. Read mapping was performed via Hisat2 (v2.1.0) using the human genome (GRCh38 v97) as a reference. Gene quantification was done via Feature Counts for raw read counts. Differentially expressed genes were identified using the edgeR (negative binomial) feature in CLCGWB (Qiagen, Redwood City, CA) using raw read counts. Genes with a *p* value <0.05, after Benjamini‐Hochberg's correction was applied, were considered significantly upregulated. Functional annotation analysis on differentially expressed genes was performed using DAVID as previously described (Huang et al., 2009). In comparative analysis, published data (Casella et al., 2019) from IMR90s made senescent with gamma radiation and serial passaging were used.

### 2.16 Relative mitochondrial membrane potential

Mitochondrial membrane potential was assessed using MitoTracker dyes: one that was potential‐dependent and one that was not, MitoTracker Red CMXRos and MitoTracker Green, respectively. Briefly, IMR90s treated for a week with vehicle or 4‐HNE were loaded with MitoTracker dyes for 45 min and fluorescence was quantified at an excitation/emission of 579 nm/599 nm and 490 nm/516 nm using a Synergy Neo 2 Multi‐mode Reader. Potential‐dependent dye signal was normalized to total mitochondrial content signal and expressed as a fold‐control of vehicle‐treated IMR90s.

### 2.17 TUNEL assay

Apoptotic phenotypes were assessed using a commercial kit to fluorescently tag 3′‐hydroxyl ends of DNA fragments (Abcam, ab66108). Briefly, IMR90s treated with vehicle and 4‐HNE continuously for 1 week were fixed with formaldehyde on ice, incubated in a FITC‐based stain targeted to DNA fragments, and then counterstained with propidium iodide to visualize all cells. Samples were then imaged on a Leica Fluorescence Microscopy system (DM5500B).

### 2.18 DNA binding assays

Commercially available DNA binding assays were used to quantify transcription factors bound to nuclear DNA (NF‐κB: Cayman 10,007,889). Briefly, nuclear extracts were prepared by lysing cells in hypotonic buffer (20 mM Tris–HCl, 10 mM NaCl, 3 mM MgCl_2_, pH 7.4) supplemented with protease inhibitors and 10% NP‐40 detergent. Nuclei were then pelleted by low‐speed centrifugation for 10 min (700 × *g* at 4°C). The pellet was rinsed once and then resuspended into hypotonic buffer and mechanically lysed through a 28‐gauge needle, yielding nuclear extracts. Samples were then added to clear plates coated with immobilized binding sequences. Plates were rinsed, probed with the antibodies, developed with chromogenic dyes, and their absorbance was measured on a Synergy 4 Plate Reader (Agilent).

### 2.19 Statistical analysis

Unpaired, two‐tailed Student's or Welch's T tests (between 2 experimental groups) were performed as appropriate. *p*‐Values below 0.05 were deemed statistically significant. All experimental groups have a sample size of at least 3, and measurements were taken from distinct biological samples. The statistical test(s) used and whether they are one‐ or two‐sided is presented in each figure legend. All data in figures are expressed as mean ± standard error of the mean (SEM).

## 3 RESULTS

### 3.1 Lipid enals induce a senescent phenotype in IMR90 fibroblasts and murine adipose stromal vascular fraction cells

Work by Curtis et al., as well as Long et al., have shown that in obese, glucose intolerant C57Bl/6J mice that the expression of the key antioxidant enzyme glutathione S‐transferase A4, as well as peroxiredoxin 3, glutathione peroxidase 4, and aldehyde dehydrogenase 2 are each down‐regulated in visceral, but not subcutaneous adipose tissue from high fat fed animals (Curtis et al., 2010; Long et al., 2013). At the cellular level using primary fat cells and 3 T3‐L1 adipocytes, inflammatory cytokines such as TNFα and IL‐1β similarly down‐regulate antioxidant gene expression (Curtis et al., 2010). The down regulation of the antioxidant enzymes in turn leads to the increased abundance of 4‐HNE and 4‐ONE in visceral, but not subcutaneous fat (Long et al., 2013). These finding suggest that lipid aldehydes accumulate in visceral, but not subcutaneous adipose tissue due to the down‐regulation of antioxidant enzymes and that inflammatory cytokines drive such processes.

Medium chain lipid enals are both soluble in aqueous systems and capable of rapidly diffusing across biological membranes making them ideal candidates for signaling molecules. Indeed, in a variety of systems 4‐HNE has been shown to diffuse into the nucleus of cells and covalently modify DNA leading to formation of N^2^‐propano‐HNE‐deoxyguanine (Douki et al., 2004), a reaction that promotes G:C to A:T substitution mutations. Additionally, 4‐HNE modifies the side chains of amino acids in proteins, particularly lysine, cysteine and histidine, leading to altered properties (Esterbauer et al., 1991). Given the increased levels of lipid enals in visceral fat, and that both DNA damage and mitochondrial dysfunction are inducers of cellular senescence, we hypothesized that adipocyte 4‐HNE and other enals such as 4‐hydroxy *trans* 2,3 hexenal and 4‐oxy *trans* 2,3 nonenal would induce senescence in cultured cells.

**FIGURE 1:**
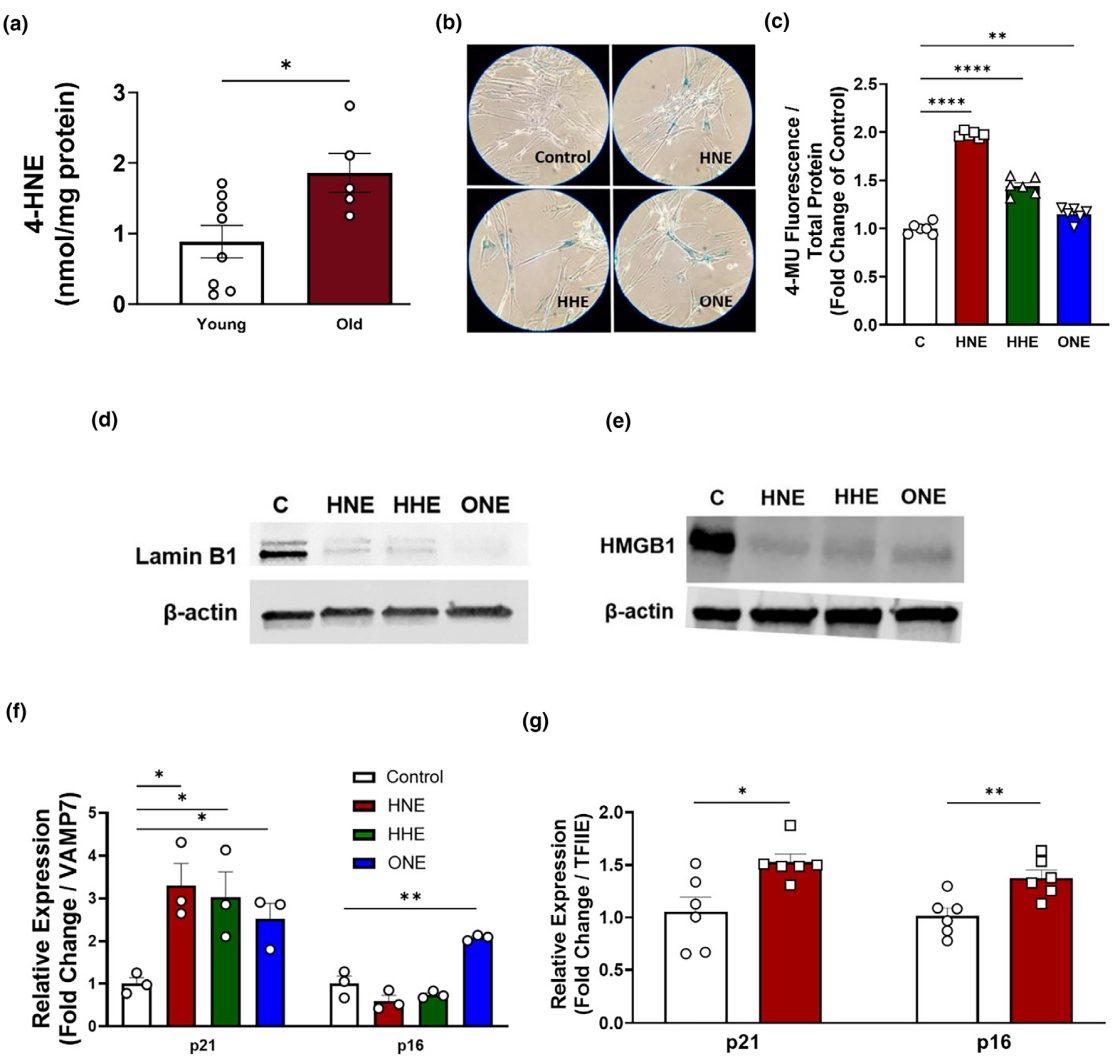
Lipid enals induce cellular senescence in IMR90s. (a) Quantitation of 4‐HNE in epididymal adipose from young (4‐month) and old (24‐month) mice by mass spectrometry. (b, c) SA‐β‐galactosidase activity in continuously treated IMR90s as indexed by chromogenic substrate X‐gal (b) and fluorogenic substrate MUG (c). (d, e) Representative immunoblots of intracellular LaminB1 (d) and HMGB1 (e) from continuously treated cells. Relative expression of p21 and p16 in continuously treated IMR90s (f) and murine SVF cells (g). *p < 0.05 versus vehicle control. Data are representative of *n* = 3–8.

To assess the physiological relevance of lipid enals to adipose tissue in advanced age, we quantified 4‐HNE in the epididymal adipose depot in mouse models of aging by mass spectrometry. Compared to young (4–6 months) mice, old mice (24–26 months) have approximately 2 times the level of 4‐HNE in epididymal white adipose tissue (EWAT) (Figure 1A). To examine whether these enals indeed induce senescence, IMR90 cells were plated and while subconfluent, treated with lipid enals for 7 days (see methods), and SnC markers then assessed including (SA‐β‐gal) activity, cell cycle arrest, loss of cellular lamin B1, loss of HMGB1, and enhanced expression of cyclin‐dependent kinase inhibitors p21^Cip1^ and p16^ink4A^. Using the fluorogenic β‐galactosidase substrates 4‐methylumbelliferyl‐β‐d‐galactopyranoside (MUG) as well as the chromogenic substrate X‐gal we found that lipid enal‐treated cells exhibited increased SA‐β‐gal activity compared to the vehicle control in a time and concentration dependent manner (Figure 1B,C, S2A,B). Gating individual cells by fluorescence intensity, we estimate that the 7‐day lipid enal treatment results in approximately 10% of cells becoming positive for SA‐β‐gal (Figure S2C,D). Lipid enal‐treated fibroblasts also exhibited slower proliferation and lost expression of lamin B1 and HMGB1 (Figure 1D,E S1A,B, S2E). All lipid enals caused a significant upregulation of expression of *CDKN1A* (p21^Cip1^), and 4‐ONE also significantly upregulated expression of *CDKN2A* (p16^ink4A^) in IMR90 cells (Figure 1F). The downregulation of p16ink4A at the transcriptional level in response to 4‐HNE was confirmed at the protein level (Figure S2F). Considered altogether, these observed changes to a suite of classic senescence biomarkers in concert indicated the initiation of the senescence program in a process we termed biogenic lipid‐induced senescence (BLIS). To confirm that BLIS occurs in cell types other than IMR90 fibroblasts, we applied continuous 4‐HNE treatment to murine stem cells isolated from the stromal vascular fraction (SVF) of visceral (perigonadal) fat and similarly measured upregulated expression of both p21^Cip1^ and p16^ink4A^ by qPCR (Figure 1G). These results are consistent with previous result demonstrating that 4‐HNE also induces biomarkers associated with senescence in human endothelial cells and skin fibroblasts, respectively (Fafián‐Labora et al., 2020; Riahi et al., 2015). Thus, lipid enals appear able to induce senescence‐like phenotypes in a variety of cell types.

### 3.2 4‐HNE causes genotoxic stress

**FIGURE 2:**
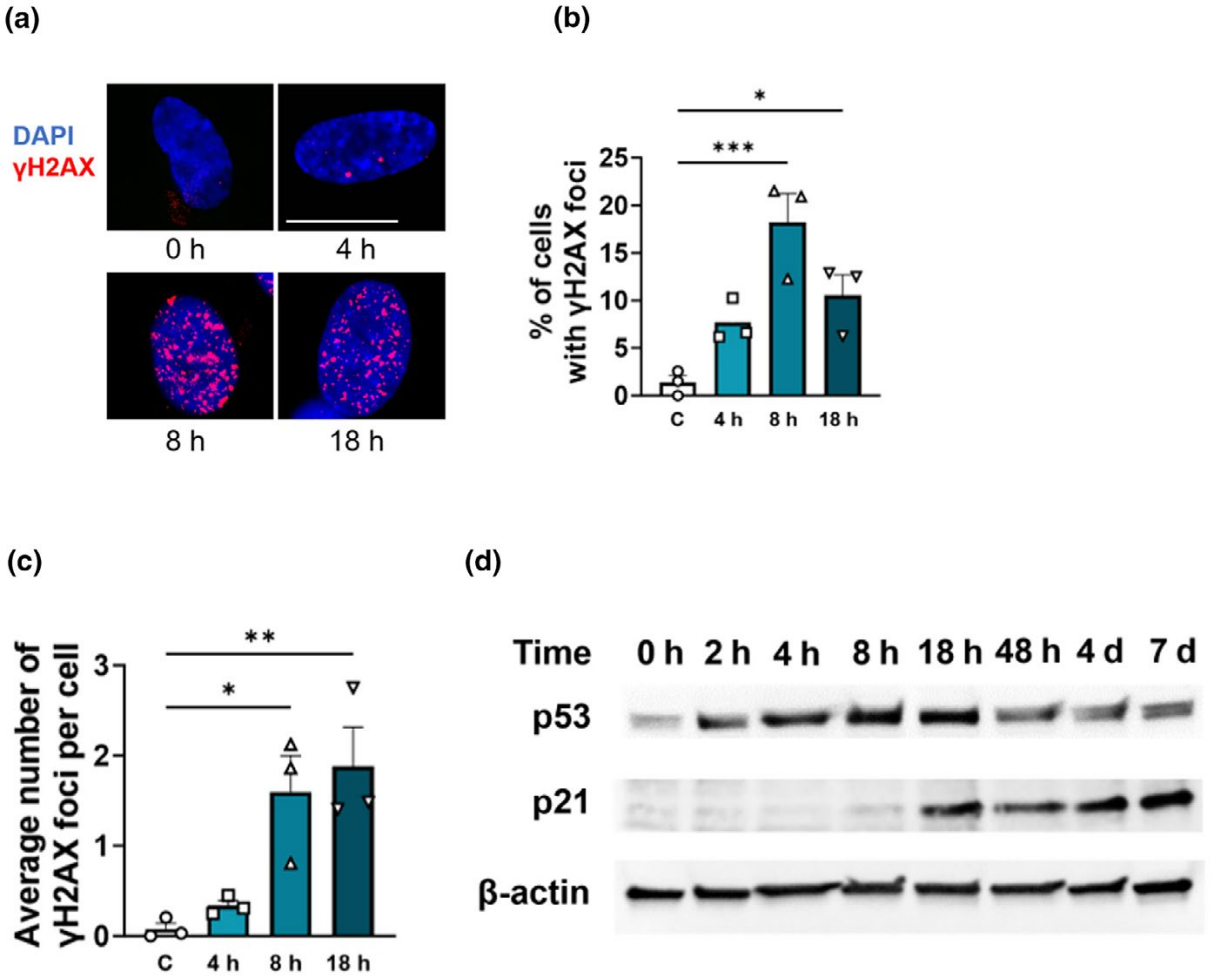
4‐HNE induces signs of genotoxicity. (a–c) Representative images of γH2AX foci in IMR90s after indicated time of 4‐HNE (20 μM) treatment and corresponding quantifications (d) Representative immunoblot of p53 and p21 after indicated time of 4‐HNE (20 μM) treatment. *p < 0.05 versus vehicle control. Scale bar is 20 μm. Data are representative of *n* = 3.

Genomic instability encompassing many types of genetic damage including telomeric attrition and double‐stranded DNA breaks, are a well‐established determinant of senescence. Similar to their reaction with the side chain amine from lysine, reactive lipid enals covalently modify the N^2^‐amine of deoxyguanine generating N^2^‐(3‐oxopropyl)‐dG. This product rearranges with the reversible modification of N^1^, giving rise to 1,N^2^ dG adducts that both hinder DNA replication and are mutagenic (Minko et al., 2009). To address possible DNA modification and subsequent double‐stranded DNA breaks with BLIS, we evaluated hallmarks of canonical γH2AX/p53/p21 pathway signaling. Concomitant with 4‐HNE treatment, the formation of γH2AX foci appeared approximately 4 h after 4‐HNE treatment, was significantly increased by 8 h. and occurred coincident with an increase in p53 (Figure 2A–C). Increased expression of p53 was followed by enhanced expression of p21^Cip1^, which remained persistently elevated even after p53 levels returned to near basal levels (Figure 2D, S1C,D). Induction of these signaling pathways suggests the creation of double‐stranded DNA breaks, potentially arising from modification of DNA.

### 3.3 Lipid enals generate protein adducts and mitochondrial dysfunction in IMR90 fibroblasts

**FIGURE 3:**
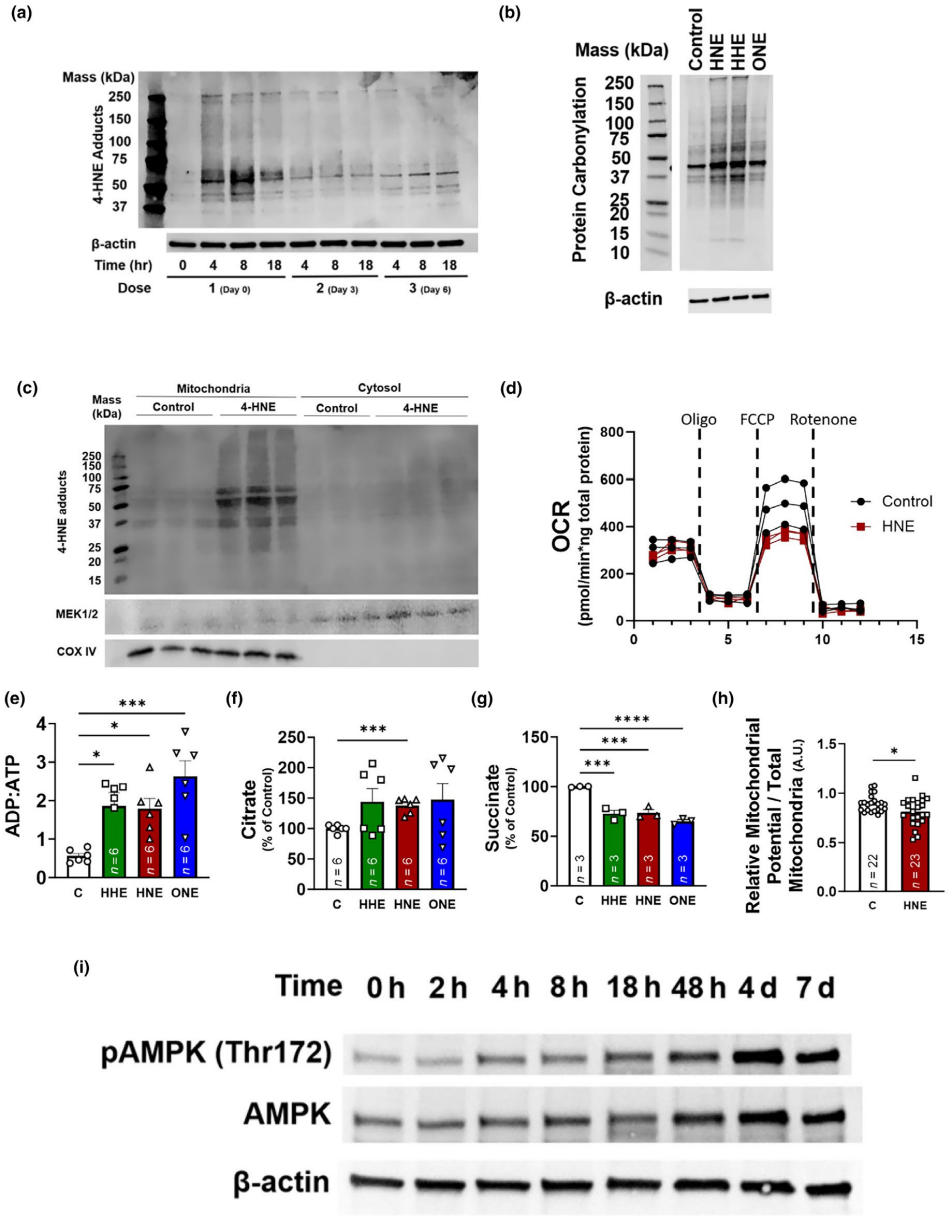
Lipid enals induce carbonyl stress and mitochondrial dysfunction in IMR90s. (a) Representative immunoblot of HNE‐protein Michael adducts formed in IMR90s with continuous treatment of 4‐HNE (20 μM). (b) Representative blot of carbonylated proteins with continuous treatment for a week with 4‐HNE, 4‐HHE, and 4‐ONE. (c) Representative immunoblot of HNE‐protein Michael adducts formed in mitochondria and cytosol after 4 h of treatment with 4‐HNE (20 μM). (d) Oxygen consumption of continuously treated IMR90s undergoing a mitochondrial stress test. (e) ADP:ATP ratios in fibroblasts treated with indicated enal continuously for 7 days. (f, g) Relative levels of citrate (f) and succinate (g) in fibroblasts treated with indicated enal continuously for 7 days. (h) Relative mitochondrial potential, normalized to total mitochondria. (i) Phospho‐ and total AMPK levels after exposure to 4‐HNE for the indicated times. *p < 0.05 versus vehicle control. Data are representative of *n* = 3–6.

The electrophilicity of enals such as 4‐hydroxy, *trans* 2,3 nonenal, makes them subject to covalent adduction with the side chains of proteins, especially those with nucleophilic amines (Esterbauer et al., 1991). One of the main reactions that lipid enals participate in is formation of Michael adducts wherein attack occurs on the β‐carbon in the lipid α, β‐unsaturated bond. Further rearrangement can result in stable hemi‐acetal groups making this covalent modification effectively irreversible (Schaur, 2003). To evaluate if treatment of IMR90 fibroblasts with 4‐HNE results in stable modification of proteins, we performed immunoblot analysis using antibodies specific for HNE‐protein adducts (Long et al., 2013). Characterization of 4‐HNE‐modified protein adducts over the course of the continuous treatment demonstrated an increase in 4‐HNE adducts 4–8 h after initial exposure after which adduct levels were maintained at a static lower‐level despite subsequent treatments with 4‐HNE on day 3 and day 6 (Figure 3A, S1E). Whereas 4‐HNE, 4‐HHE, and 4‐ONE appear to modify the same set of proteins, 4‐HNE and 4‐HHE modified proteins were more abundant relative to modification with 4‐ONE (Figure 3B, S1F). Fractionating the 4‐HNE modified proteins revealed that the majority of HNE‐induced protein carbonylation were mitochondrial rather than cytoplasmic (Figures 3C, S3A).

Given the capacity of oxidized enals to modify proteins, they can induce mitochondrial dysfunction via loss of function of TCA cycle and electron transport chain constituents such as aconitase, Complex I, Complex III and the ATP Synthase (Curtis et al., 2012). To assess mitochondrial function in 4‐HNE‐treated IMR90 cells, we utilized Seahorse technology to quantify oxygen consumption in response to enal challenge. IMR90 cells exhibited reduced mitochondrial spare capacity following a weeklong exposure to 4‐HNE (Figures 3D, S3B), along with an increase in ADP and a decrease in ATP levels (Figures 3E, S3I–K), consistent with a decrease in energy charge (Figure S3K). 4‐HNE treatment also affected the tricarboxylic acid cycle with a decrease in succinate and an increase in citrate levels, suggesting potential modification of aconitase, a known 4‐HNE target (Figure 3F,G). 4‐HNE treatment reduced the mitochondrial membrane potential indicating altered mitochondrial function in cells exposed to lipid enals (Figure 3H). Phosphorylation of AMP kinase at threonine 172, a putative consequence of disrupted mitochondrial function, occurred 48 h post 4‐HNE exposure (Figures 3I, S1G). A trending increase in extracellular acidification (Figure S3C) suggests that BLIS causes SnCs to become more glycolytic, consistent with the enrichment of glycolysis‐associated genes often measured in senescent cells. Indeed, GAPDH is upregulated by nearly 2‐fold in IMR90s subjected to BLIS (Table S3). Also consistent with mitochondrial dysfunction, genes associated with fatty acid oxidation such as *HADHB* and *CPT1A* were upregulated (Table S3). Taken together, these results suggest a bimodal mechanism of BLIS involving both genotoxicity and mitochondrial damage.

### 3.4 BLIS is associated with an NF‐κB‐independent SASP

**FIGURE 4:**
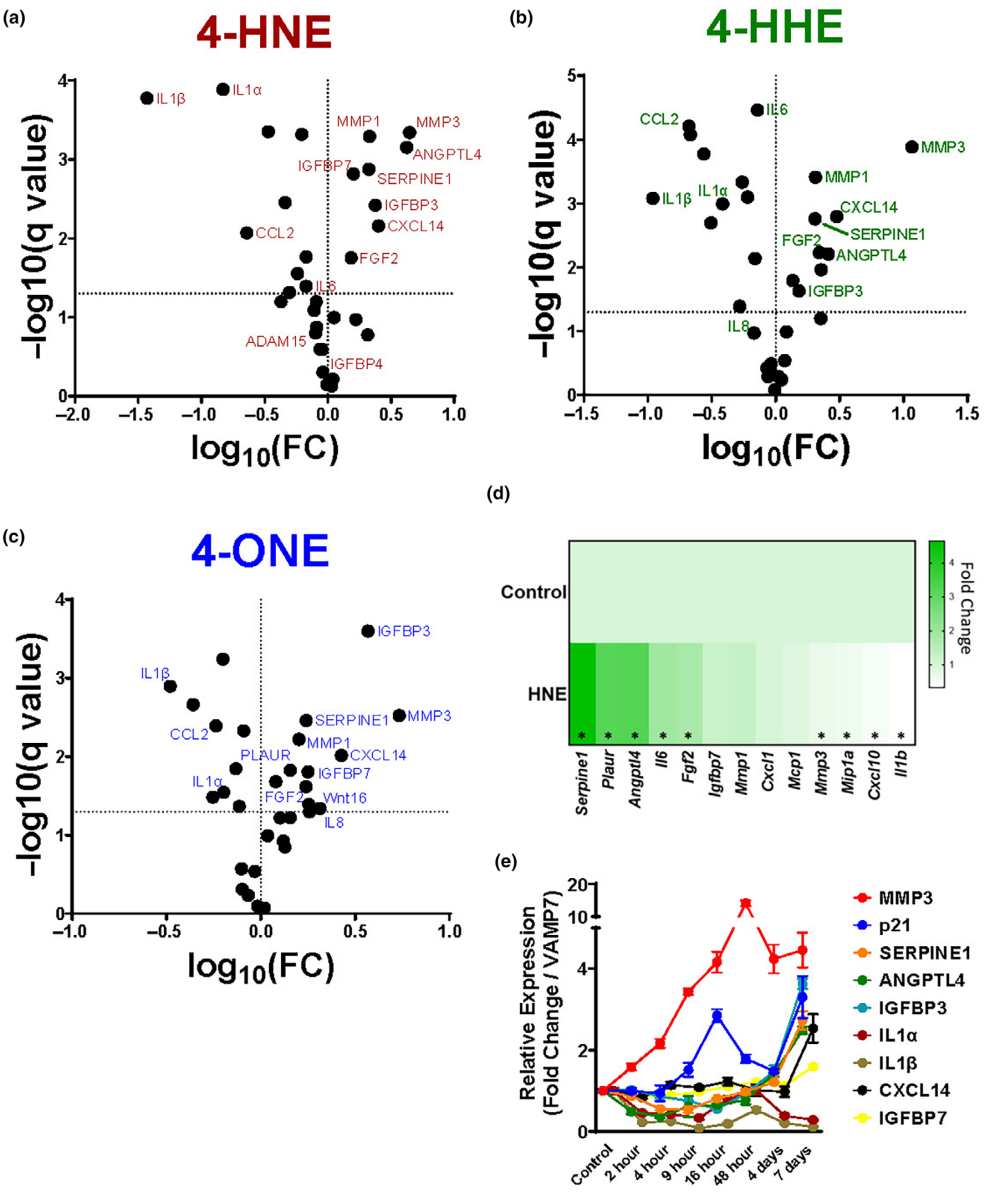
Lipid enals induce an NF‐κB‐independent portion of the SASP. (a–c) Volcano plots of expression of selected putative human SASP constituents in IMR90s after a week of continuous treatment with lipid enals (a) 4‐HNE, (b) 4‐HHE, and (c) 4‐ONE. (d) Expression of selected putative SASP constituents in murine SVF cells after a week of continuous treatment. (e) Expression of selected BLIS‐associated SASP factors in IMR90s over the course of continuous treatment at indicated times. *p < 0.05 versus vehicle control. Data are representative of *n* = 3–6.

A major feature of the senescent program is the senescence associated secretory phenotype (SASP), the composition of which varies by cell type and means of senescence induction (Sturmlechner et al., 2021). Using a candidate‐gene approach, we found that BLIS was accompanied by increased expression of several SASP constituents as defined by the SenMayo panel of factors (Figure 4A–C) (Saul et al., 2022). Among these were extracellular matrix regulators *SERPINE1* and *MMP3*, insulin‐like growth factor binding proteins 3 and 7, and growth factors *ANGPTL4* and *FGF2*. *CXCL14*, a chemokine that initiates immunosurveillance of p21^Cip1^‐positive SnC by macrophages (Sturmlechner et al., 2021), is also a member of the lipid enal‐induced SASP. Secretion of IGFBP3 and MMP3 in response to lipid enals was confirmed by ELISA measurements of target protein levels in conditioned media (Figure S4A,B).

In contrast to the upregulated SASP components, the expression of SASP constituents modulated by NF‐κB signaling such as *CXCL1* and *IL‐1β* declined upon treatment with 4‐HNE or 4‐HHE (Figure 4A,B). Intriguingly, the expression of some major NF‐κB targets such as *TIMP1*, *IL6*, and *IL8* were decreased following 4‐HNE treatment while upregulated with 4‐ONE (Figure 4C). NF‐κB inhibition by 4‐HNE treatment was previous reported in cultured chondrocytes through modification of IKKα (Vaillancourt et al., 2007). Consistent with this, 4‐HNE treatment of human IMR90 fibroblasts resulted in a decrease in NF‐κB DNA binding activity, suggesting that lipid enals decrease expression of certain SASP components via decreased NF‐κB signaling (Figure S4C). To evaluate if the secretory phenotype associated with BLIS was similar in different cell types, we measured mRNA expression of SASP factors in murine SVF cells from visceral (perigonadal) fat depots of C57BL/6J mice. The SASP from enal‐induced murine adipose stem cells largely, but not completely, phenocopied that from human fibroblasts (Figure 4D). Of particular note, a putative senescent cell surface marker Urokinase Plasminogen Activator Surface Receptor (*PLAUR*) (Amor et al., 2020) is robustly upregulated by over 3‐fold in murine SVF cells exposed to 4‐HNE while only upregulated ~10% in IMR90 cells similarly treated.

The kinetics of expression of SASP factors was not uniform for all target genes **(**Figure 4E). Upon exposure to 4‐HNE, *MMP3* mRNA expression increased immediately while expression of p21^Cip1^ was delayed until 9 h posttreatment. In contrast, *CXCL14* and *IGFBP7* required essentially 7 days of continuous 4‐HNE treatment before upregulation. Interestingly, *ANGPTL4*, *IGFBP3*, and *SERPINE1* were initially downregulated following 4‐HNE treatment before increasing above basal levels after 4–7 days (Figure 4E). These temporal differences in the emergence of secreted factors suggests a complex program of regulatory events and multiple transcription factors mediating the BLIS transcriptome.

To characterize the transcriptome that accompanies the induction of the BLIS program further, we performed RNA‐Seq analysis on 4‐HNE treated and vehicle control treated IMR90s (Table S3). Functional annotation analysis of the nearly 4500 genes upregulated in BLIS highlighted several interesting patterns: (1) transcripts for factors associated with stress response, chaperone proteins, proteasomal machinery, ubiquitin mediated proteolysis, and autophagy were all enriched (Table 1); (2) transcripts for proteins associated with DNA replication, chromosome partitioning, and cell division were depleted (Table S4); and (3) genes associated with mitochondrial proteins are upregulated while those associated with nuclear proteins are downregulated (Table 1, Table S4). Evaluation of the SASP factors by SignalP predicted that almost 60% of the proteins did not contain conventional secretion sequences and may be secreted by unconventional pathways (Teufel et al., 2022) (Figure S4D).

**TABLE 1:** DAVID Analysis of Genes Upregulated in IMR90s subject to BLIS.

Category	Term	Count	%	FDR
Biological Process	Electron transport	60	1.5	1.4E‐14
	Respiratory chain	40	1	7.1E‐12
	ER‐Golgi transport	46	1.2	4.1E‐09
	Protein biosynthesis	60	1.5	6.10E‐08
	Lipid metabolism	193	4.9	1.30E‐06
	Tricarboxylic acid cycle	17	0.4	2.20E‐05
	Apoptosis	143	3.6	3.50E‐05
	Ubl conjugation pathway	184	4.6	3.90E‐05
	Autophagy	54	1.4	8.60E‐05
	Hydrogen ion transport	26	0.7	1.30E‐04
	Lipid biosynthesis	54	1.4	3.50E‐04
	Fatty acid metabolism	50	1.3	1.30E‐03
	Glycolysis	17	0.4	2.70E‐03
	Stress response	36	0.9	4.90E‐03
	Lipid transport	46	1.2	3.90E‐02
	Fatty acid biosynthesis	19	0.5	3.90E‐02
	Carbohydrate metabolism	29	0.7	4.90E‐02
Cellular Component	Mitochondrion	476	12	2.20E‐60
	Cytoplasm	1291	32.6	1.20E‐35
	Mitochondrion inner membrane	142	3.6	6.80E‐24
	Golgi apparatus	269	6.8	1.10E‐14
	Proteasome	36	0.9	3.00E‐13
	Endoplasmic reticulum	326	8.2	2.90E‐10
	Mitochondrion outer membrane	55	1.4	2.50E‐07
	Lysosome	96	2.4	6.70E‐04
	Membrane	1547	39.1	3.50E‐03
Molecular Function	Transferase	468	11.8	1.10E‐10
	Ribosomal protein	70	1.8	2.40E‐08
	Chaperone	78	2	6.80E‐08
	Ribonucleoprotein	91	2.3	1.50E‐06
	Initiation factor	29	0.7	1.50E‐06
	Oxidoreductase	153	3.9	6.90E‐06
	Hydrolase	381	9.6	8.00E‐06
	Kinase	171	4.3	5.80E‐04
	Elongation factor	14	0.4	3.50E‐03
	Isomerase	43	1.1	7.90E‐03
	Lyase	46	1.2	2.10E‐02
	Translocase	31	0.8	2.10E‐02

*Note*: Count refers to number of upregulated genes in category, % refers to percentage of upregulated genes in category, FDR refers to *p*‐value after Benjamini‐Hochberg correction.

To identify SASP genes common across different human senescence models, we cross‐referenced our dataset with two other published datasets on ionizing radiation‐induced senescence and replicative senescence (Casella et al., 2019; Sturmlechner et al., 2021) focusing on those with at least a 2‐fold change in expression. This analysis revealed 17 common SASP factors (*OSTN*, *KISS1*, *SERPINI1*, *KRT34*, *REN*, *DKK2*, *AQP1*, *CDNF*, *WNT16*, *MFAP5*, *SEMA3E*, *BMP3*, *LIPG*, *EGFL7*, *INA*, *ULBP2*, *CTF1*), many of which are associated with extracellular matrix regulation (e.g., serine protease inhibitor I1 and renin) (Figure S4E).

To discover potential cell signaling pathways conserved across senescence induction models, we used a more inclusive dataset for IR‐induced senescence (Casella et al., 2019) and considered results meeting a false discovery rate threshold of 0.05 to be upregulated. Our analysis yielded 2017 genes that were upregulated during BLIS, IR senescence, and replicative senescence. As expected, KEGG pathway analysis revealed significant enrichment of transcripts associated with p53 signaling and cellular senescence. Functional annotation analysis identified significant enrichment of genes associated with stress response, mainly heat shock proteins, as well as the proteasome (Table S5). We also found that mitochondrial proteins and metabolic enzymes are overrepresented among the BLIS upregulated genes (Table 1). Additionally, transcripts for enzymes that participate in lipid metabolism, particularly those involved in phospholipid remodeling such as *LCLAT1*, *LIPG*, *PLA2G15*, and *PRDX6*, are represented among the common gene sets. These findings suggest that a disruption in normal protein folding and homeostasis, mitochondrial and metabolic stress, and phospholipid remodeling are features of cellular senescence that occur irrespective of what initiates the senescence.

### 3.5 BAX channel formation is required for part of the BLIS phenotype

**FIGURE 5:**
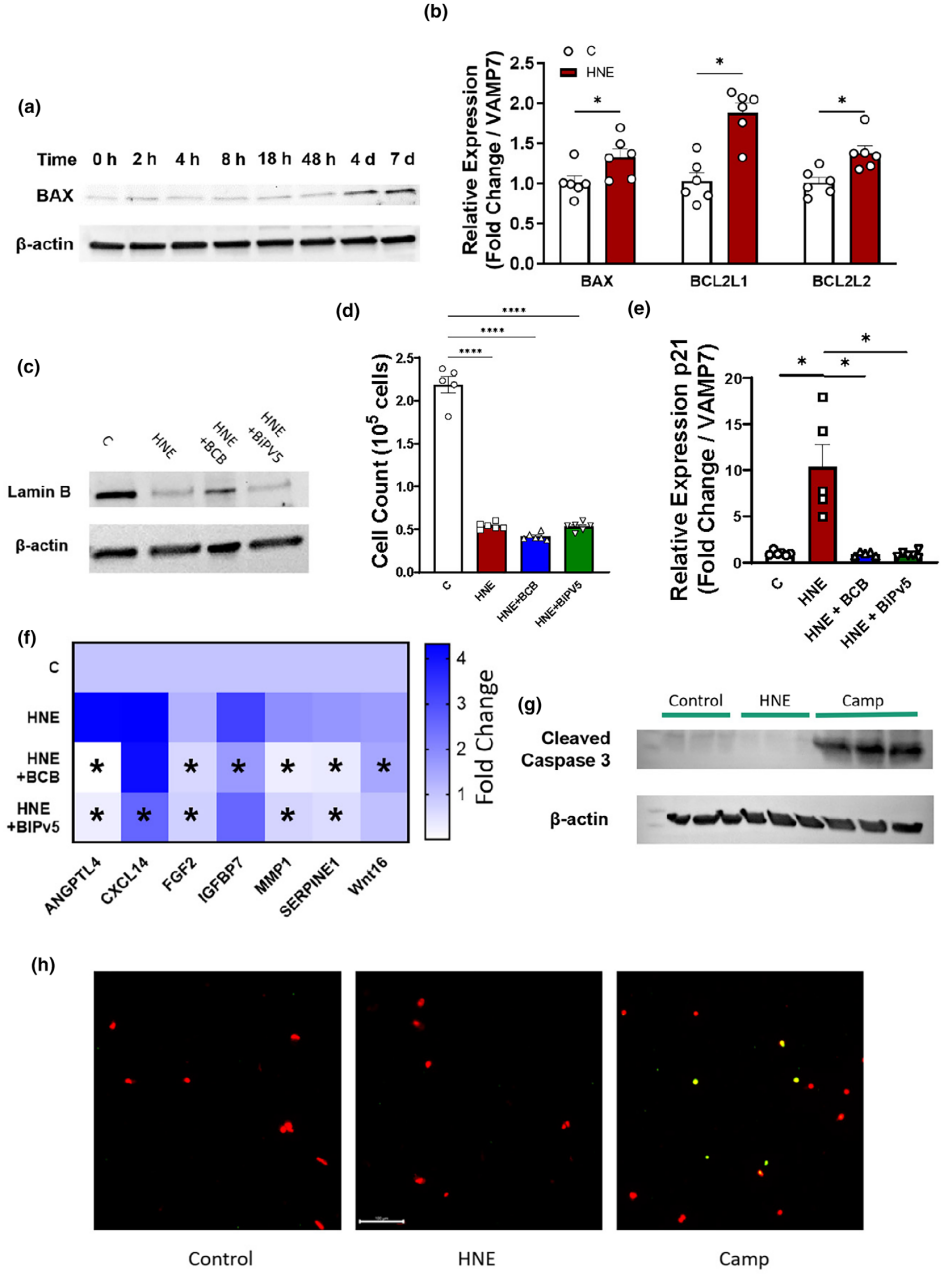
BAX signaling is necessary for BLIS (a) Representative immunoblot of relative BAX levels after indicated time of 4‐HNE exposure (b) Expression of *BAX*, *BCL2L1*, and *BCL2L2* after a week of 4‐HNE treatment. (c–f) Representative immunoblot of (c) Lamin B1, (d) cell count, (e) p21 expression, (f) BLIS factor expression after 1 week of continuous exposure to cells cotreated with 4‐HNE and BAX inhibitors as stated. *p < 0.05 versus HNE group for panel 5F. (g) Immunoblot of cleaved caspase 3 after 7‐day 4‐HNE treatment. (h) Representative images of TUNEL‐stained fibroblasts following treatment. Camptothecin used as a positive apoptotic control. All experiments performed on IMR90 fibroblasts. *p < 0.05. Data are representative of *n* = 3.

Passos and colleagues have proposed that sublethal apoptotic stress is a determining feature of cellular senescence (Victorelli et al., 2023) BAK/BAX macropores cause mitochondrial outer membrane permeabilization (mtMOMP), a major disruption of normal mitochondrial architecture that ends with release of mtDNA into the cytosol. As shown in both radiation‐associated and replicative senescence, when MOMP occurs in a small number of mitochondria, it leads to senescence rather than apoptosis by a processes termed minority MOMP (miMOMP) (Victorelli et al., 2023). At both the protein and mRNA levels, 4‐HNE treatment increased BAX expression; RNA‐seq and subsequent functional annotation analysis revealed a significant enrichment of genes associated with apoptosis including the anti‐apoptotic factors *BCL2L1* (Bcl‐xL) and *BCL2L2* (Bcl‐w) (Figure 5A,B; Table 1; Table S3). Treatment of IMR90 cells with two different BAX macropore inhibitors, BCB or BIPv5, attenuated the upregulation of p21^Cip1^ and several SASP factors by 4‐HNE without effecting loss of Lamin B1 and mitotic arrest associated with BLIS (Figure 5C–F). Furthermore, predicted activation of IRF3, a transcription factor triggered by presence of mtDNA in the cytoplasm, suggests that 4‐HNE induces mtMOMP as part of its mode of action and that mitochondrial dysfunction may be upstream of DNA damage based signaling (Figure 4F). It is important to note that 4‐HNE‐treated IMR90 cells are not themselves apoptotic. Fibroblasts that stained positive for SA‐β‐galactosidase activity exhibited a spread morphology associated with senescence rather than a compact, rounded morphology characteristic of apoptosis and fibroblasts treated with lipid enals over a week were TUNEL negative and did not exhibit an accumulation of cleaved caspase 3 (Figures 1A, 5G,H, S1I).

### 3.6 L‐carnosine attenuates the senescent phenotype brought on by lipid enals

**FIGURE 6:**
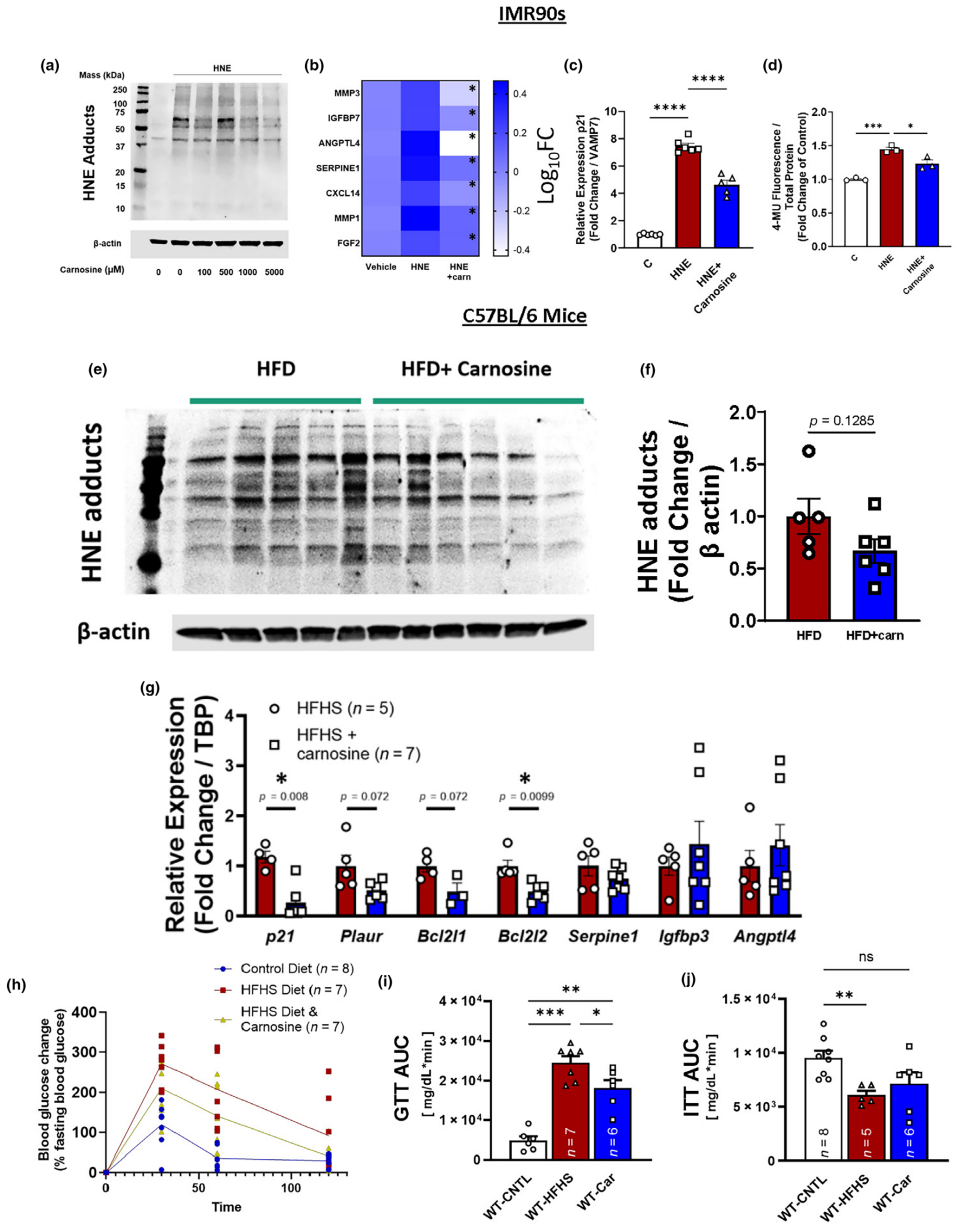
Carnosine attenuates the senescent phenotype (a) Representative immunoblot of 4‐HNE adducts in HNE‐treated IMR90 cells with indicated concentration of L‐carnosine. (b–d) Expression of select BLIS factors, *p < 0.05 compared to HNE alone (b), SA‐β‐galactosidase activity indexed by MUG (c), and expression of p21 (d) in IMR90s concurrently treated with 4‐HNE and L‐carnosine. (e, f) Immunoblot and accompanying quantitation of 4‐HNE Michael adducts in epididymal white adipose tissue (EWAT) of C57BL/6 mice treated with a high fat diet (HFD) with and without L‐carnosine (80 mM in drinking water). (g) p21 and selected BLIS signature constituents in EWAT from high fat high sucrose (HFHS) fed mice treated with L‐carnosine (80 mM in drinking water). (h) Glucose tolerance test (GTT) of normal chow diet (control) fed mice and HFHS‐fed mice with and without carnosine. (i, j) Areas under the curves of glucose (GTT) and insulin tolerance tests (ITT) of mice fed specified diets. *p < 0.05. Data are representative of *n* = 3–6.

L‐carnosine is a dipeptide of histidine and beta‐alanine that is most abundant in tissue with high mitochondrial content such as the heart and brain. As a Michael acceptor, carnosine forms an covalent adduct with 4‐HNE (Aldini et al., 2002) both in situ and in vivo, and its capacity to scavenge a variety of other biogenic aldehydes has been well documented (Palmer et al., 2019). Concurrent treatment of IMR90 cells with L‐carnosine in the presence of 4‐HNE exposure resulted in a reduction of HNE adduct formation (Figures 6A, S1J), attenuation of BLIS as measured by expression of *MMP3*, *IGFBP7*, *ANGPTL4*, *SERPINE1*, *CXCL14*, *MMP1*, and *FGF2* (Figure 6B), as well as reduced SA‐β‐galactosidase activity and p21^Cip1^ expression (Figure 6C,D).

In visceral adipose from C57BL/6J mice, obesity is associated with increased carbonyl stress (Long et al., 2013) and expression of senescence markers, particularly p21^Cip1^ (Wang et al., 2022). To address the potential of 4‐HNE and other bioactive lipid enals to be inducers of cellular senescence in vivo, we maintained mice on a high fat or high fat, high sugar diet in the presence or absence of carnosine and evaluated 4‐HNE Michael adducts in EWAT SVF, glucose tolerance, and the expression of senescence markers in EWAT. Treatment of HFD‐fed mice with L‐carnosine resulted in a downward trend in 4‐HNE Michael adducts in the SVF of EWAT, suggesting that carnosine is, in fact, scavenging endogenous 4‐HNE (Figure 6E,F). Importantly, carnosine treatment significantly blunted the expression of *p21* ^*Cip1*^ and *Bcl2l2* and the expression of other senescence‐associated transcripts *Plaur* and *Bcl2L1* trended downward (Figure 6G). Concomitant with a downregulation in *p21* ^*Cip1*^ was an improvement in glucose tolerance (Figure 6H,I), but no change in insulin tolerance (Figure 6J). Administration of carnosine to HFD‐fed mice did not lead to a change in overall body weight (Figure S4F).

## 4 DISCUSSION

Reactive lipid enals containing activated double bonds such as 4‐HNE, 4‐HHE, and 4‐ONE are products of lipid peroxidation that readily react with nucleophilic species on proteins and nucleic acids (Esterbauer et al., 1991). Studies by Long et al., 2013 demonstrated that lipid enals are formed most avidly in the visceral, but not subcutaneous, depots of white adipose tissue in response to obesity. In parallel, work by Xu et al. (Aldini et al., 2002) and Palmer et al., 2019 demonstrated that white adipose tissue accumulated SnCs in response to high fat feeding and that genetic or pharmacologic ablation of such cells improved metabolic dysfunction and attenuated characteristics of age‐dependent processes (Aldini et al., 2002; Xu, Palmer, et al., 2015). Despite the strong correlative link between visceral obesity and senescence, the molecular mechanisms that link these together has been enigmatic. Our in vitro findings are consistent with the hypothesis that biogenic lipid enals such as 4‐HNE, 4‐HHE, or 4‐ONE are inducers of cellular senescence. In support of this hypothesis, we used human IMR90 or murine adipose stem cells to demonstrate that (1) lipid enals induce hallmarks of DNA damage and cell cycle arrest including upregulation of p53, p21^Cip1^, and γH2AX foci; (2) 4‐HNE modifies cellular proteins, particularly mitochondrial proteins, supporting mitochondrial dysfunction and limited mitochondrial outer membrane permeabilization in the absence of apoptosis; (3) lipid enals induce an NF‐κB independent arm of the human SASP characteristic of primary senescence; and (4) scavenging biogenic aldehydes can ameliorate BLIS both in vitro and in vivo. Consistent with the concept that biogenic aldehydes induce senescence in vivo, comparison of 15 life‐extending interventions found overexpression of the major 4‐HNE detoxification enzyme, GstA4, in 9 interventions, making it the most frequently shared feature of these geroprotectors (Tyshkovskiy et al., 2019). Moreover, the ability to metabolize endogenously‐generated aldehydes has been found to be correlated with lifespan in *C elegans* models where loss of Gsta4 function shortened lifespan while Gsta4 over expression lengthened lifespans (Ayyadevara et al., 2007). Taken together, our results and previous studies demonstrate the potential for biogenic enals to initiate and/or potentiate cellular senescence.

Reactive lipid enals have been shown to activate some biomarkers of senescence in a limited number of in vitro models (Fafián‐Labora et al., 2020; Riahi et al., 2015; Swiader et al., 2021). Importantly, a common theme is that the senescence inducer directly or indirectly increases production of reactive oxygen species. Studied stressors included sublethal doses of H_2_O_2_ along with several organic peroxide species and ethanol (Toussaint et al., 2000). Our results recapitulate previous observations that 4‐HNE and 4‐ONE initiate the senescence program, extends that finding to the related lipid enal 4‐HHE, and characterize the associated senescence programming. Studies of 4‐HHE largely phenocopied those of 4‐HNE in the transcriptional profile of cyclin‐dependent kinase inhibitors and SASP factors while 4‐ONE deviated from that with several key markers including *p16* ^*ink4a*^, *IL6*, and *IL8*. Due to the presence of a strongly electron withdrawing carbonyl group, 4‐ONE has been characterized as much more reactive than the 4‐hydroxy enal lipids (Lin et al., 2005). Such a difference in reactivity may translate to differences in protein and/or DNA modification and therefore, differentially affect senescence programming (Aldini et al., 2002). Future detailed proteomic studies should provide more granular detail on differential modification between 4‐HNE and 4‐ONE and downstream effects on the senescence phenotype.

These studies suggest that biogenic lipid enals affect cellular senescence through mechanisms involving both genetic damage and mitochondrial dysfunction incurred after alkylation (Figures 2, 5). Mitochondrial dysfunction and DNA double‐stranded breaks enacted by pharmacological means are known to induce cellular senescence, and we observed evidence of each occurring after 4‐HNE treatment (Wiley et al., 2016). An important cell signaling pathway involved in senescence initiation is p53 signaling, known to play a role in both the cellular response to genotoxicity and mitochondrial dysfunction associated senescence (MiDAS) (Victorelli et al., 2023). Our results are consistent with both an immediate p53 response to the oxidative insult of 4‐HNE and chronic p53 signaling more characteristic of MiDAS. Recently, it has been proposed that sublethal apoptotic stress mediates the development of senescence (Victorelli et al., 2023) by BCL2 signaling leading to BAX polymers to form pores in the mitochondrial membrane, facilitating mtMOMP. Progressively wider BAX pores in the mitochondrial membranes permit the release of mtDNA into the cytosol, and through cGAS‐STING signaling mediates the SASP. Our finding that mitochondrial stress response and apoptotic programming are shared features of BLIS support the hypothesis that persistent, low‐level stress underpins cellular senescence and its accompanying secretory phenotype.

Comparing the transcriptome of 4‐HNE‐induced IMR90 senescence with that of replicative senescence and/or ionizing radiation‐induced IMR90 senescence revealed commonalities that warrant future study. Remodeling of cardiolipin, a lipid found specifically in the mitochondria, has been found to drive 4‐HNE generation and redox dysfunction during myocardial infarction (Jia et al., 2021). Indeed, ALCAT1 (*LCLAT1*), the acyltransferase responsible for this adverse remodeling, was upregulated in BLIS (Table S3). Among the upregulated factors shared between radiation senescence, replicative senescence, and BLIS is peroxiredoxin 6 (Prdx6), a dual‐functioning enzyme that has both glutathione peroxidase and phospholipase A2 activity (Fisher, 2011). Prdx6 is upregulated in tBHP‐associated senescence (Dierick et al., 2002) where it mediates the extracellular matrix regulation arm of the SASP (Salovska et al., 2022). Notably, we found that an extracellular matrix‐regulated portion of the SASP, including serine protease inhibitor *SERPINI1*, is also conserved across the types of senescence we analyzed.

Sequestering biogenic aldehydes as a therapeutic strategy to mitigate senescence‐associated pathologies has previously shown potential (Hipkiss et al., 2016). Fafian‐Labora et al. used an aging mouse model to demonstrate that small extracellular vesicles from young fibroblasts alleviate senescence‐related markers through glutathione S transferase activity (Fafián‐Labora et al., 2020). Moreover, Swiader et al. used L‐carnosine to blunt the formation of γH2AX foci and restore SIRT1 expression in fibroblasts exposed to UV‐A radiation (Swiader et al., 2021). Consistent with these observations, we found L‐carnosine to be effective in partially ameliorating the senescent phenotype brought on by lipid enals in vitro. Scavenging 4‐HNE prevented upregulation of SA‐β‐galactosidase activity, p21^Cip1^ expression, and expression of SASP factors in cultured fibroblasts subjected to BLIS. In parallel, we treated diet‐induced obese C57BL/6J mice with 80 mM L‐carnosine in the drinking water for 8 weeks and measured blunted expression of *CDKN1A* (p21^Cip1^) and *Bcl2L2* in the stromal vascular fraction of visceral fat, a critical finding given that high p21^Cip1^ expression in visceral adipose depots drives metabolic dysfunction (Wang et al., 2022) while Bcl2 proteins facilitate the MOMP. Additionally, L‐carnosine treatment blunts the expression of *Plaur*, a cell surface marker of senescent cells and a target for CAR T senolytic therapy (Amor et al., 2020). A concomitant decrease in 4‐HNE‐protein adducts in the stromal vascular fraction of the visceral adipose depot suggests a connection between the biogenic lipid‐derived electrophiles and senescence in adipose. Further study should be undertaken on L‐carnosine and more bioavailable derivatives to further evaluate the therapeutic potential of scavenging endogenously‐generated enals and aldehydes.

A limitation of our cell‐based studies is that all measurements were made in bulk using cultured cell systems. Our in vitro analysis indicates that 4‐HNE treatment of cultured cells results in both genotoxic and proteotoxic stress, but not which one (or both), is required for induction of the senescence phenotype. Moreover, the studies were carried out in cell lines that may contain primary and secondary senescence subpopulations expressing different gene expression profiles. Although bulk measurements still yielded sufficient resolution to observe classic senescence hallmarks, future studies in animal models utilizing single cell transcriptomics, spatial analysis, and proteomics will yield more granular information on mechanisms underlying BLIS. In addition, while L‐carnosine treatment of experimental mice attenuates the adipose BLIS phenotype, there remain multiple potential mechanisms beyond simple adduction of 4‐HNE including amplification of endogenous L‐carnosine synthesis and activation of antioxidant signaling pathways. Further in vivo studies will be needed to elucidate the relevant mechanisms at play in lipid‐induced senescence.

## AUTHOR CONTRIBUTIONS

TBM developed hypothesis, carried out experiments, wrote and edited the manuscript. AVM developed the hypothesis, carried out experiments, wrote and edited the manuscript. DMD carried out experiments and edited the manuscript. TH carried out experiments and edited manuscript. SVS carried out experiments and edited the manuscript. IB carried out experiments and edited the manuscript. CH carried out experiments and edited the manuscript. PP carried out experiments, wrote, and edited the manuscript. EJA developed the hypothesis, edited the manuscript. CDC developed the hypothesis and edited the manuscript. PDR developed the hypothesis and edited the manuscript. DAB developed the hypothesis, wrote, and edited the manuscript.

## CONFLICT OF INTEREST STATEMENT

None declared.

## Supporting information


Figure S1.

Figure S2.

Figure S3.

Figure S4.

Table S1.

Table S2.

Table S3.

Table S4.

Table S5.

## Data Availability

Raw RNA sequencing data contained in Table S3 are available from the NCBI under BioProject ID PRJNA1048426. All raw data are available from corresponding author DAB (bernl001@umn.edu) upon reasonable request.
