# Supplementary figures and images for: Lipid peroxidation products induce carbonyl stress, mitochondrial dysfunction, and cellular senescence in human and murine cells
Source: Aging Cell. 2024 Oct 11;24(1):e14367. doi: 10.1111/acel.14367 (PMC11709094; doi:10.1111/acel.14367)

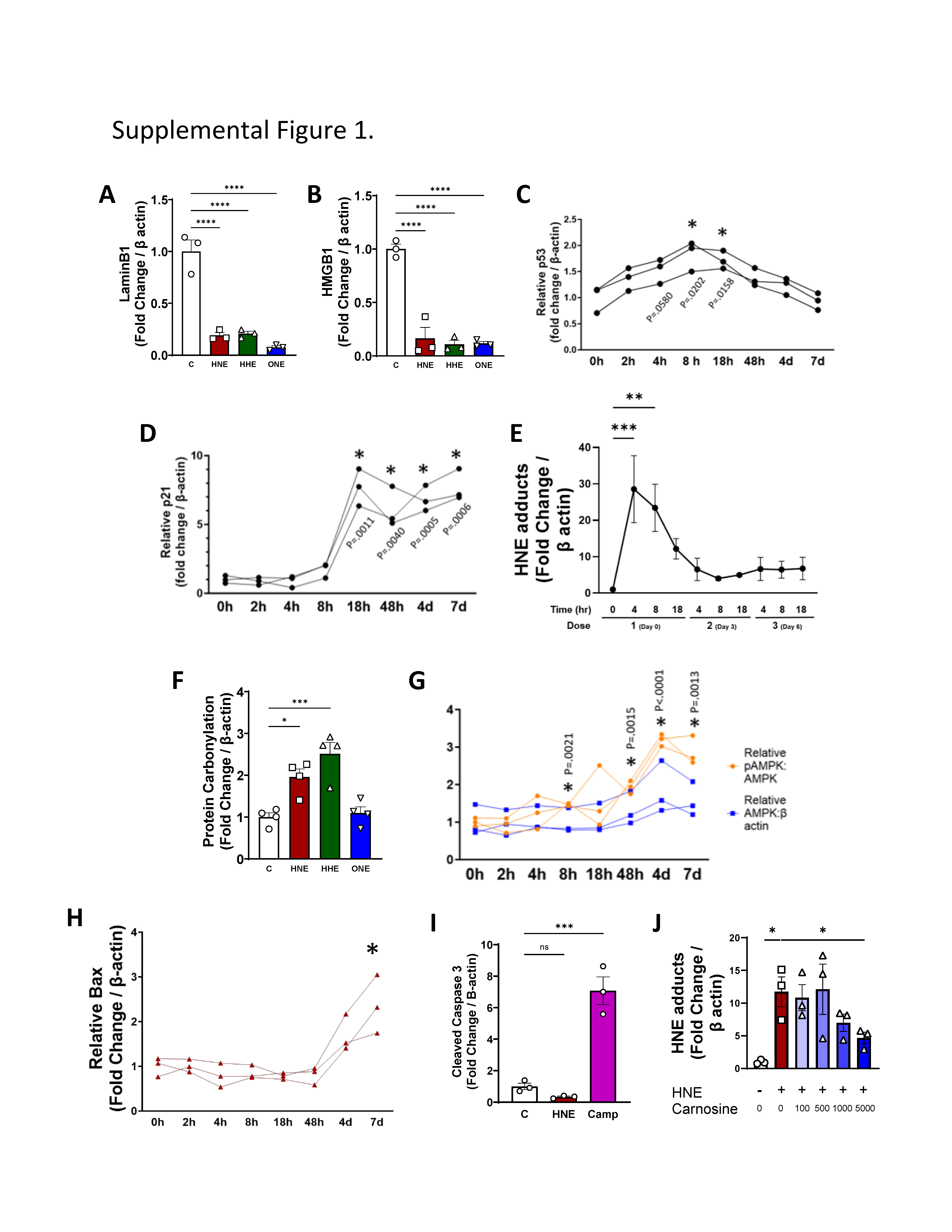

Supplement: Supplementary file 1 — Figure S1. [file ACEL-24-e14367-s008.jpg]

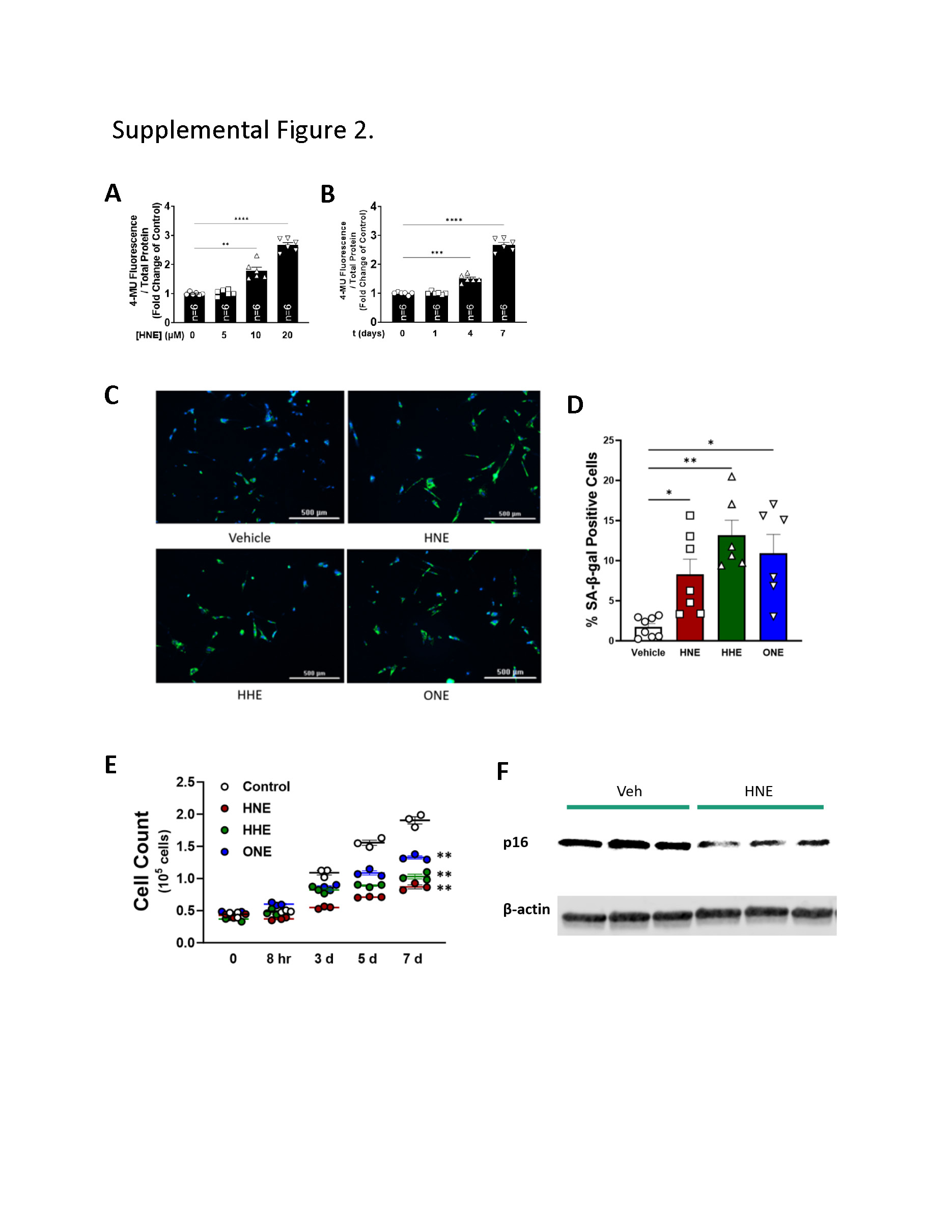

Supplement: Supplementary file 2 — Figure S2. [file ACEL-24-e14367-s007.jpg]

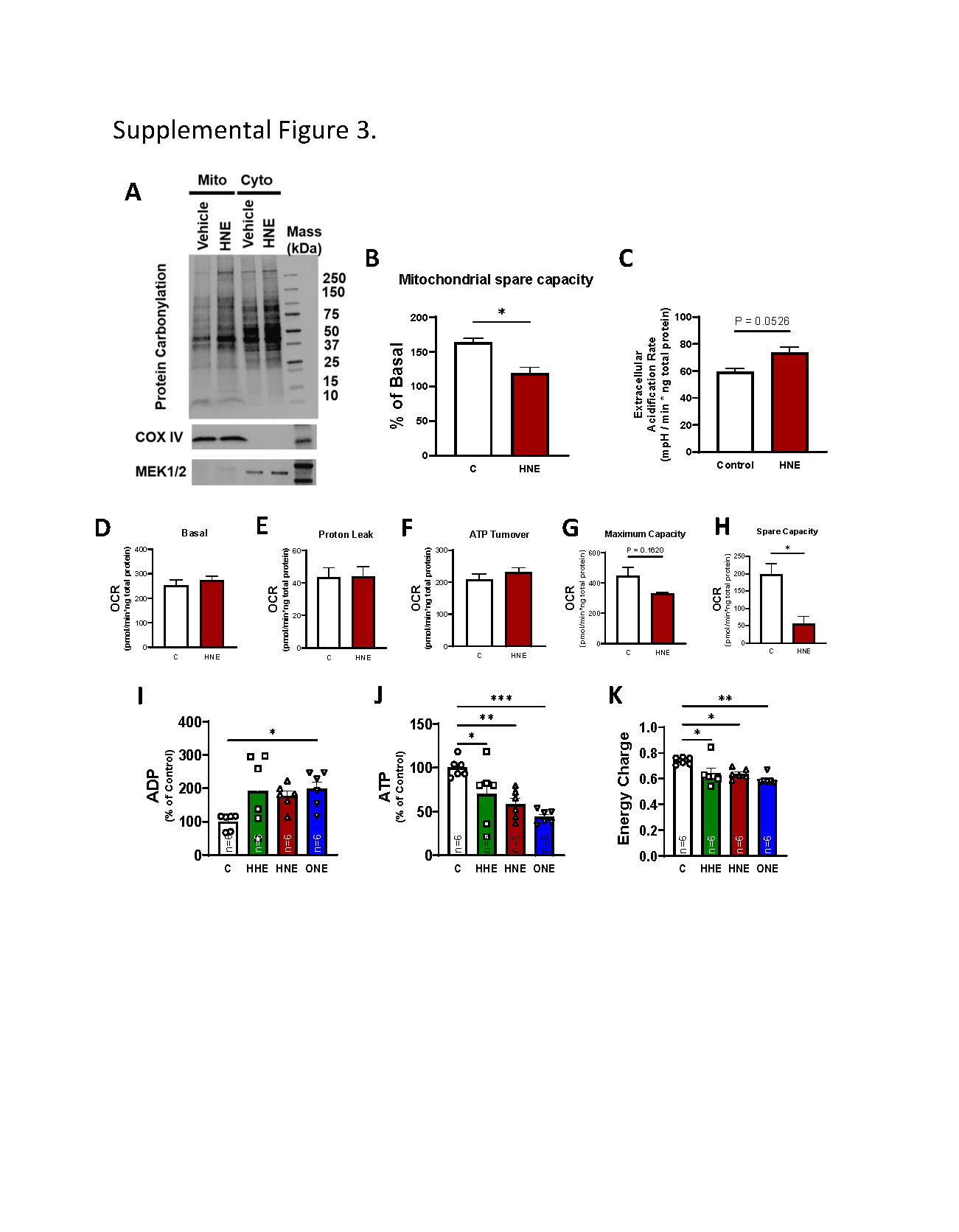

Supplement: Supplementary file 3 — Figure S3. [file ACEL-24-e14367-s003.jpg]

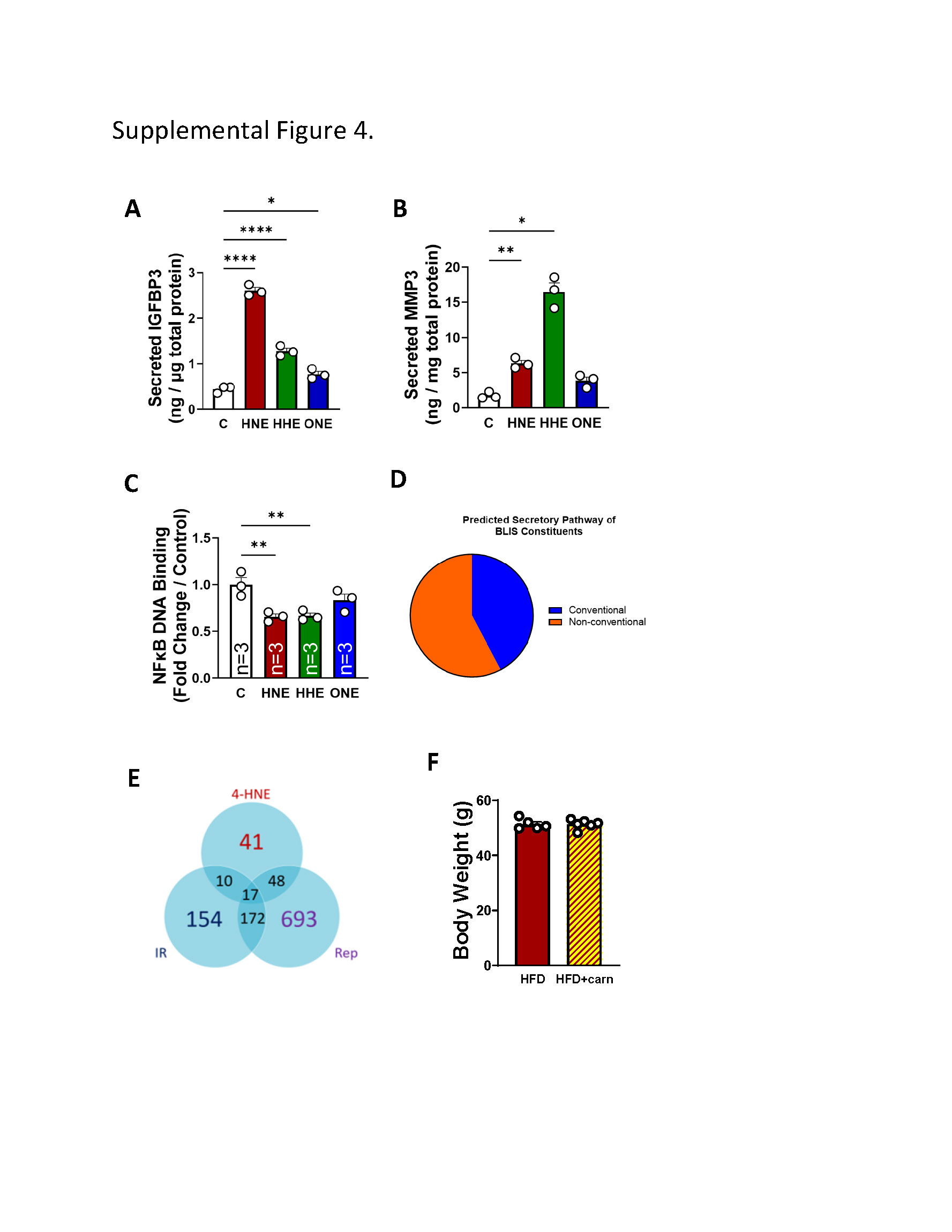

Supplement: Supplementary file 4 — Figure S4. [file ACEL-24-e14367-s006.jpg]
