# Supplementary material for: Lipid peroxidation products induce carbonyl stress, mitochondrial dysfunction, and cellular senescence in human and murine cells
Source: Aging Cell. 2024 Oct 11;24(1):e14367. doi: 10.1111/acel.14367 (PMC11709094; doi:10.1111/acel.14367)
Supplement: Supplementary file 5 — Table S1. [file ACEL-24-e14367-s002.docx]

**Supplemental Table 1.** Reagents used in this study.

| **Species** | **Target** | **Supplier, Catalogue Number** | **Application** | **Dilution** |
| --- | --- | --- | --- | --- |
| Rabbit | Reduced HNE Michael Adducts | Sigma-Aldrich, 393207 | WB | 1:1000 |
| Rabbit | Lamin B1 | Abcam, ab16048 | WB | 1:1000 |
| Rabbit | HMGB1 | Abcam, ab18256 | WB | 1:1000 |
| Mouse | p53 | EMD-Millipore, 05-224 | WB | 1:1000 |
| Rabbit | p21 Waf1/Cip1 | Cell Signaling, 2947 | WB | 1:1000 |
| Rabbit | Phospho-AMPKα (Thr172) | Cell Signaling, 2535 | WB | 1:500 |
| Rabbit | AMPKα | Cell Signaling, 2532 | WB | 1:1000 |
| Mouse | β-Actin | Sigma-Aldrich, A2228 | WB | 1:1000 |
| Rabbit | Cleaved Caspase 3 | Cell Signaling, 9664 | WB | 1:1000 |
| Rabbit | Phospho-Histone H2A.X (Ser139) | Cell Signaling, 9718 | ICC | 1:200 |
| Mouse | BAX | Invitrogen, MA514003 | WB | 1:300 |
|  |  |  |  |  |
